# *In vitro* evolution of durlobactam resistance in NDM-producing *Escherichia coli* due to a single mutation in *mrdA* encoding penicillin-binding protein 2

**DOI:** 10.1128/aac.01014-25

**Published:** 2025-10-10

**Authors:** Christi L. McElheny, Erika L. Butcher, Akito Kawai, Robert M. Q. Shanks, Ryan K. Shields, Yohei Doi

**Affiliations:** 1 Division of Infectious Diseases, University of Pittsburgh School of Medicine 271847, Pittsburgh, Pennsylvania, USA; 2 Department of Microbiology, Fujita Health University School of Medicine 89305, Toyoake, Aichi, Japan; 3 UPMC Eye Center, University of Pittsburgh School of Medicine 12317 , Pittsburgh, Pennsylvania, USA; 4 Center for Innovative Antimicrobial Therapy, University of Pittsburgh School of Medicine 12317 , Pittsburgh, Pennsylvania, USA; 5 Antibiotic Management Program, University of Pittsburgh Medical Center 6595, Pittsburgh, Pennsylvania, USA; University of Fribourg, Fribourg, Switzerland

**Keywords:** durlobactam, penicillin-binding protein, β-lactam inhibitor

## Abstract

Durlobactam, a diazabicyclooctane β-lactamase inhibitor, exhibits direct antibacterial activity by binding to penicillin-binding protein 2 (PBP2). We generated a mutant strain of New Delhi metallo-β-lactamase-producing *Escherichia coli* with a durlobactam minimum inhibitory concentration of 2 µg/mL, representing a 16-fold increase from baseline, by exposing it to increasing concentrations of durlobactam. Resistance was attributed to a point mutation in the *mrdA* gene, resulting in a V522I substitution in PBP2.

## INTRODUCTION

Multidrug-resistant gram-negative bacteria pose a growing threat to global health. A key mechanism underlying their resistance is the production of β-lactamases, which are enzymes that inactivate β-lactam antibiotics. Among these, the spread of New Delhi metallo-β-lactamase (NDM)-producing *Escherichia coli* strains has become a significant clinical challenge. NDM confers resistance to a broad range of β-lactam antibiotics, including carbapenems, complicating the treatment of infections. Current guidelines recommend treating infections caused by NDM-producing *E. coli* with cefiderocol or ceftazidime–avibactam in combination with aztreonam (1). These antibiotics work in part by inhibiting penicillin-binding protein 3 (PBP3). However, mutations in PBP3 in NDM-producing *E. coli* have been reported to cause resistance to these antibiotics (2–5), further leading to challenges in treatment.

New β-lactam/β-lactamase inhibitor combinations have shown promise in combating multidrug-resistant infections. Durlobactam, a novel diazabicyclooctane (DBO) β-lactamase inhibitor, is active against a broad spectrum of β-lactamases, including Class A, C, and D enzymes, with particular activity against acquired OXA carbapenemases produced by *Acinetobacter baumannii* (6). Durlobactam was recently approved by the U.S. Food and Drug Administration in combination with sulbactam, an older β-lactamase inhibitor with direct activity against *A. baumannii* PBP3, for the treatment of hospital-acquired and ventilator-associated pneumonia caused by susceptible *A. baumannii* strains (7).

Resistance mechanisms to sulbactam–durlobactam in *A. baumannii*, including mutations in PBP3 and altered efflux or porin expression, have been described (6, 8). Durlobactam also inhibits penicillin-binding protein 2 (PBP2) in Enterobacterales, exhibiting antibacterial activity, but resistance mechanisms in Enterobacterales have not been characterized (9). Here, we investigated the mechanisms underlying durlobactam resistance in an NDM-producing *E. coli* strain.

An *E. coli* clinical strain (NDM-Parent), with a minimum inhibitory concentration (MIC) of 0.125 µg/mL, was used to generate *in vitro* mutants. NDM-Parent is an *E. coli* ST95 strain isolated from a patient urine specimen in 2013 (10). The strain was serially passaged in Mueller–Hinton broth containing increasing concentrations of durlobactam at 37°C with shaking. Overnight cultures were started in the presence of no durlobactam and were transferred daily into fresh media with increasing concentrations, starting at two times the MIC (0.25 µg/mL), and increased twofold until no growth was observed, which occurred at 4 µg/mL. This *in vitro* mutation experiment was performed twice, and one culture from each experiment resulted in growth at increased concentrations of durlobactam. These mutant strains (NDM-S5 and NDM-S6) showed a 16-fold increase in durlobactam MIC, reaching 2 µg/mL (Table 1).

**TABLE 1:** Durlobactam MICs for the *E. coli* parent, *mrdA* mutant, and recombinant strains^*a*^

Strain	Characteristics	Durlobactam MICs (µg/mL)
NDM-Parent	Wild type	0.125
NDM-S5	*In vitro* generated *mrdA* mutant (V522I)	2
NDM-S6	*In vitro* generated *mrdA* mutant (V522I)	2
NDM-Parent_V522I	Allelic recombinant of parent strain with mutated *mrdA* gene from NDM-S5	2

^*a*^ MIC, minimum inhibitory concentration.

Whole genome sequencing with Illumina followed by Breseq analysis (version 0.37.1) revealed a mutation in *mrdA*, which encodes PBP2, resulting in a valine-to-isoleucine substitution at position 522 (V522I) in both mutants. Additional mutations were identified in a transposase gene and in the intergenic region of a hypothetical protein. In addition, NDM-S6 harbored a T107S substitution in the FimF protein, a component of type 1 fimbriae along with FimA, FimG, and FimH, which together constitute bacterial adhesion structures of *E. coli* (11).

To determine whether the PBP2 V522I substitution conferred increased durlobactam MICs, we introduced the mutation into the NDM-Parent strain via recombineering (12). Electrocompetent parent cells containing plasmid pMQ763, which carries a hygromycin resistance marker and an L-arabinose-inducible lambda red recombinase system, were transformed with a 1,171 bp *mrdA* gene product that contained the mutation, amplified using the following primers: 5′-AATCTGGCCGATTTTACGCTC-3′ and 5′-GCTAAGGCCAGAGAGGAACA-3′. Recombinants were selected on lysogenic broth (LB) agar plates containing 0.25% L-arabinose, 600 µg/mL hygromycin, and 2 µg/mL durlobactam. Colonies were subsequently passaged on non-selective LB agar plates to facilitate curing of pMQ763. Single colonies were then passaged on LB agar plates with 600 µg/mL hygromycin. Colonies that failed to grow were presumed to have lost the pMQ763 plasmid and were selected for amplification of the *mrdA* region and then sent for Sanger sequencing. A successful recombinant that contained the V522I mutation (NDM-Parent_V522I) was identified.

The recombinant strain NDM-Parent_V522I also exhibited a durlobactam MIC of 2 µg/mL, demonstrating that the V522I mutation alone decreases susceptibility to durlobactam compared with the parent strain. When tested in a fixed-ratio broth microdilution assay, the strains with V522I displayed a fourfold increase in MIC to sulbactam–durlobactam. We additionally tested the strains against avibactam, the first approved DBO β-lactamase inhibitor, to assess cross-resistance. The V522I substitution resulted in an eightfold increase in MIC of ceftazidime–avibactam (Table 2).

**TABLE 2:** MICs of the *E. coli* parent, *mrdA* mutant, and recombinant strains to additional antibiotics and β-lactamase inhibitors^*a*^

	MICs (µg/mL)						Fixed ratio^*b*^	
	SUL	DUR	SUD^*c*^	CAZ	AVI	CZA^*c*^	CZA	SUD
NDM-Parent	>128	0.125	NG	>128	16	>128	24/16	0.5/0.5
NDM-S5	>128	2	NG	>128	>128	>128	192/128	2/2
NDM-Parent_V522I	>128	2	NG	>128	>128	>128	192/128	2/2
ATCC25922	32	0.25	NG	0.25	16	0.25	0.75/0.5	0.5/0.5

^*a*^ AVI, avibactam; CAZ, ceftazidime; CZA, ceftazidime–avibactam; DUR, durlobactam; MIC, minimum inhibitory concentration; NG, no growth; SUD, sulbactam–durlobactam; SUL, sulbactam.

^*b*^ The fixed ratios were 3:2 for ceftazidime:avibactam and 1:1 for sulbactam:durlobactam.

^*c*^ The concentration of durlobactam and avibactam was a fixed concentration of 4 μg/mL in the SUD and CZA combinations.

**Fig 1:**
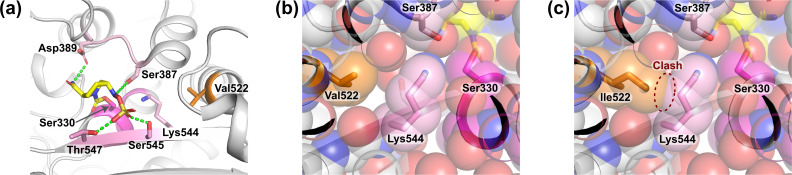
Modeled binding of avibactam to the active site of *E. coli* PBP2 with V522I substitution. The figures were generated using the crystal structure of the *E. coli* PBP2–avibactam complex (PDB ID: 6G9F [13]), with the V522I substitution introduced via the mutagenesis tool in PyMOL (The PyMOL Molecular Graphics System, version 3.0; Schrödinger, LLC). (**a**) Avibactam (yellow) is covalently bound to active-site Ser330 (magenta), highlighting key stabilizing interactions with residues Ser387, Asp389, Ser545, and Thr547 (pink). Hydrogen bonds are represented as green dashed lines. (**b and c**) Close-up view of the Lys544 residue. Transparent space-filling models indicate the van der Waals radii of the respective atoms. (**b**) Crystal structure. (**c**) V522I model, suggesting that the substituted Ile522 (orange) introduces a steric clash with Lys544 (pink).

To explore structural implications, we modeled the V522I mutation using the previously reported crystal structure of *E. coli* PBP2 bound to avibactam (PDB ID 6G9F, Fig. 1) (13). Although avibactam lacks the double bond and methyl side chain present in durlobactam, it shares the core scaffold and binding mode, including covalent interaction with the active-site Ser330 and key hydrogen bonds to surrounding residues, namely, Asp389 via its amide group, Ser387 via the nitrogen adjacent to its sulfate, and Ser545 and Thr547 via its sulfate moiety (Fig. 1a). As the experimental structure of durlobactam-bound PBP2 is not yet available, this avibactam-based model was used for discussion. The V522I substitution likely induces subtle conformational changes within PBP2 that affect its active site geometry. While Val522 is located far from the active site, it lies in close proximity to Lys544, a residue that precedes Ser545 in the sequence and is spatially adjacent to Ser330 (Fig. 1b). Lys544 is buried within the molecule, suggesting that the bulkier isoleucine introduced by the V522I substitution may require a slight structural rearrangement to alleviate steric hindrance (Fig. 1c). This structural rearrangement may reduce the carbamoylation efficiency of durlobactam either by altering the positioning of the nucleophilic Ser330 or by altering the spatial orientation of Lys544, thereby disrupting the hydrogen-bonding network among Ser545, Thr547, and the substrate-binding groove. These findings align with previous reports where small structural rearrangements in PBPs caused significant reductions in β-lactam susceptibility, underscoring the importance of allosteric effects in resistance evolution (14).

A key question is whether the V522I mutation imposes a biological cost on bacterial fitness, such as reduced growth rate, morphological defects, or compromised cell wall integrity. Although this study did not assess these parameters directly, prior work on *mrdA* has demonstrated that resistance-conferring mutations can result in peptidoglycan remodeling or impaired septation, depending on the structural context (15). Investigating whether the V522I mutation alters PBP2’s catalytic activity or substrate specificity could provide critical insight into the evolutionary constraints acting on resistance pathways.

Mutations in PBP2, alone or in combination with other mutations, have previously been linked to decreased susceptibility to other DBO β-lactamase inhibitors (15–18). Notably, the V522I substitution was recently linked to decreased susceptibility to cefepime–zidebactam in NDM-producing *E. coli* (19), but its role in durlobactam resistance has not been described. Increasing clinical use of PBP2-active DBOs like durlobactam may select for such structural alterations in PBP2, undermining the activity of β-lactam-DBO combinations against difficult-to-treat strains they are intended to target. Further studies are warranted to evaluate the clinical impact of PBP2 mutations on resistance to DBOs.
